# Comparing the effectiveness of magnesium oxide and naldemedine in preventing opioid-induced constipation: a proof of concept, single institutional, two arm, open-label, phase II, randomized controlled trial: the MAGNET study

**DOI:** 10.1186/s13063-020-04385-0

**Published:** 2020-06-01

**Authors:** Anna Ozaki, Takaomi Kessoku, Michihiro Iwaki, Takashi Kobayashi, Tsutomu Yoshihara, Takayuki Kato, Yasushi Honda, Yuji Ogawa, Kento Imajo, Takuma Higurashi, Masato Yoneda, Masataka Taguri, Takeharu Yamanaka, Hiroto Ishiki, Noritoshi Kobayashi, Satoru Saito, Yasushi Ichikawa, Atsushi Nakajima

**Affiliations:** 1 Department of Gastroenterology and Hepatology, Yokohama City University Graduate School of Medicine, 3-9 Fukuura, Kanazawa-ku, Yokohama, 236-0004 Japan; 2 Department of Palliative Care Center, Yokohama City University Hospital, 3-9 Fukuura, Kanazawa-ku, Yokohama, 236-0004 Japan; 3 Department of Gastroenterology, International University of Health and Welfare Atami Hospital, 13-1 Higashikaigan-cho, Atami, 413-0012 Japan; 4 Department of Biostatistics, Yokohama City University Graduate School of Medicine, 3-9 Fukuura, Kanazawa-ku, Yokohama, 236-0004 Japan; 5 Department of Palliative Medicine, National Cancer Center Hospital, Tokyo, 104-0045 Japan; 6 Department of Oncology, Yokohama City University Hospital, 3-9 Fukuura, Kanazawa-ku, Yokohama, 236-0004 Japan

**Keywords:** Opioid-induced constipation, Magnesium oxide, Naldemedine, Randomized controlled trial

## Abstract

**Background:**

Patients taking opioids are known to develop opioid-induced constipation (OIC), which reduces their quality of life. The aim of this study is to compare magnesium oxide with naldemedine and determine which is more effective in preventing OIC.

**Methods:**

This proof-of-concept, prospective, randomized controlled trial commenced in Japan in March 2018. Initially, a questionnaire-based survey will be conducted targeting adult patients with cancer who concomitantly commenced opioid treatment and OIC prevention treatment. Patients will then be randomly allocated to a magnesium oxide group (500 mg thrice daily) or a naldemedine group (0.2 mg once daily). Each drug will be orally administered for 12 weeks. The primary endpoint is defined as any improvement in scores on the Japanese version of Patient Assessment of Constipation Quality of Life questionnaire (JPAC-QOL) from baseline to 2 weeks of treatment.

**Discussion:**

The primary endpoint is change in JPAC-QOL score from baseline to 2 weeks of intervention. The key secondary endpoint will be change in spontaneous bowel movements at 2 and 12 weeks of intervention. This study will determine whether magnesium oxide or naldemedine is more effective for the prevention of OIC.

**Trial registration:**

University Hospital Medical Information Network (UMIN) Clinical Trials Registry, UMIN000031891. Registered March 25, 2018.

## Background

Opioids are used for cancer pain management [1, 2]; however, there are challenges associated with continuous opioid therapy, owing to complications such as nausea, constipation, sleepiness, and respiratory depression [3–6]. Constipation develops in 15–64% of patients receiving strong opioid analgesics [7–11], and chronic constipation may occur more frequently in women (male/female ratio, 1:2.2) and in older persons [12]. In patients with various cancers in Japan, the cumulative incidence of opioid-induced constipation (OIC) is lung, 48%; pancreatic, 53%; colon, 60%; breast, 79%; stomach, 71%; esophageal, 60%, prostate, 50%; bladder, 50%; and others, 59% [13]. Long duration of opioid therapy is largely responsible for OIC [14], and drug tolerance against OIC is rarely established, so preventive administration of laxatives is important [15].

Symptoms of constipation (abdominal pain, fullness, and loss of appetite) impair patients’ quality of life (QOL); thus, OIC is a problem worth investigating. Traditional OIC treatment involves either nondrug therapy comprising consumption of high-fiber diets or the administration of medications such as laxatives. In Japan, the Clinical Guidelines for Gastrointestinal Symptoms in Cancer Patients recommend osmotic laxatives [16]. A Japanese observational study reported that preventive magnesium oxide intake attenuated OIC when patients commenced opioid therapy [17]. Thus, osmotic laxatives including magnesium oxide are a conventional OIC treatment in Japan. OIC occurs when opioids act on μ-receptors on intestinal nerves, reducing intestinal motility and fluid secretion [6, 18]. Both nondrug treatments and osmotic laxatives do not target the underlying mechanism of OIC [3, 9].

Over the years, little progress has been made in OIC treatment research [9]. Recently, peripherally acting μ-opioid receptor antagonists (PAMORAs) were shown to be effective in treating OIC. Naldemedine is a novel PAMORA being developed for the treatment of OIC without affecting central analgesia [19]. Furthermore, its safety and efficacy have been reported to be superior to placebo [20, 21]. Patients with OIC sometimes feel irritated, stressed, and uncomfortable because of their restricted diet, or they are ashamed of their frequent and long bathroom breaks, especially during social activities. Constipation impairs patients’ QOL; hence, there is a need for preventive treatment. This study will compare magnesium oxide with naldemedine and determine which is more effective in preventing OIC.

## Methods

### Trial design

This study is a proof-of-concept, single-institution, two-arm, open-label, phase II, randomized controlled trial comparing the effectiveness of magnesium oxide (500 mg thrice daily) with that of naldemedine (0.2 mg once daily) to prevent OIC for 12 weeks (MAGNET study). The primary endpoint will be the change in the Japanese version of the Patient Assessment of Constipation Quality of Life questionnaire (JPAC-QOL) score from baseline to 2 weeks of treatment. The study aims to recruit 120 adult patients with cancer from the Yokohama City University Hospital cohort.

**Fig. 1:**
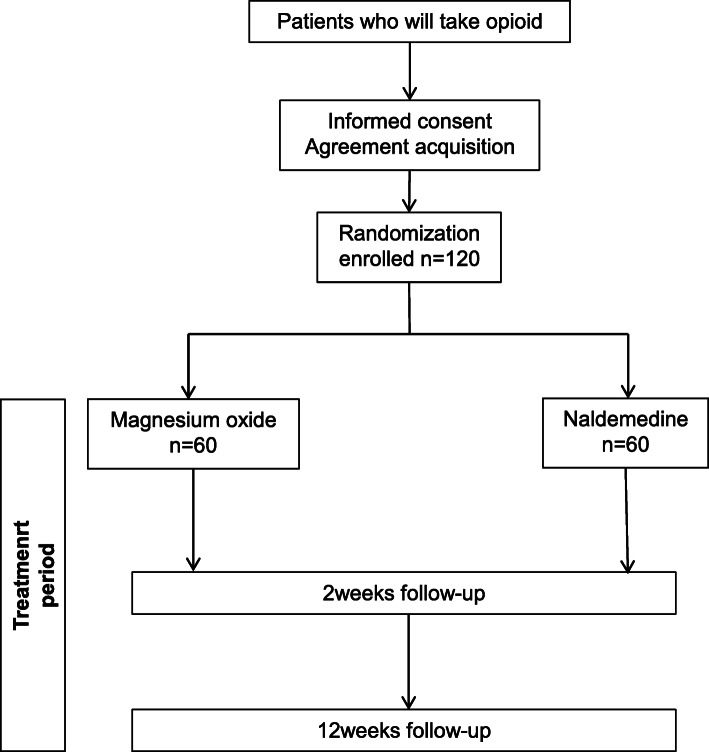
Study flowchart

**Fig. 2:**
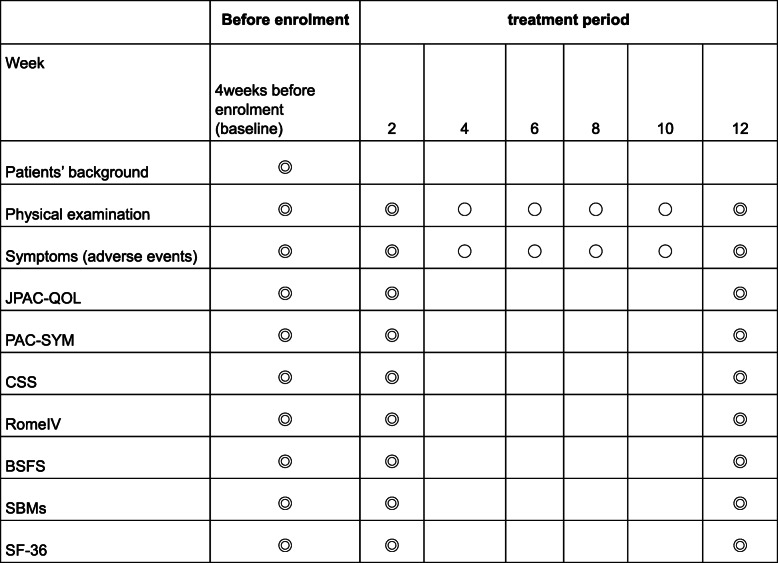
Study schedule. All objectives will be compared between magnesium oxide and naldemedine. *BSFS* Bristol Stool Form Scale, *CSS* Constipation Scoring System, *JPAC-QOL* Japanese version of Patient Assessment of Constipation Quality of Life questionnaire, *PAC-SYM* Patient Assessment of Constipation Symptoms, *SBMs* Spontaneous bowel movements, *SF-36* 36-item Short Form Health Survey

A flowchart of the study is shown in Fig. 1. Evaluations will be performed at three time points: at baseline and 2 and 12 weeks after intervention, as shown in Fig. 2.

### Ethical issues

The study will be performed in accordance with the Declaration of Helsinki principles and the Japanese ethical guidelines for clinical research. The protocol was approved by the Ethics Committee of Yokohama City University Hospital on March 22, 2018. The Standard Protocol Items: Recommendations for Interventional Trials patient-reported outcome extension and its checklists were followed in preparing the protocol. This trial is registered in the University Hospital Medical Information Network (UMIN) Clinical Trials Registry under identifier UMIN000031891. All participants will be required to provide written informed consent. The protocol and any information supplied to gain informed consent were approved by the qualified Institutional Review Board/Independent Ethics Committee of Yokohama City University prior to patient enrollment. The participants’ personal information will be maintained in a separate locked cabinet and password-protected hard drive at Yokohama City University. Records will be retained for 5 years after study completion and then destroyed by the data center.

### Study endpoints

The expected endpoints are listed in Table 1. The primary endpoint is the change of JPAC-QOL from baseline to 2 weeks with magnesium oxide versus naldemedine intervention. JPAC-QOL consists of 28 questions assessed using a 5-point adjectival score from 1 to 5, with a lower score indicating a better outcome for QOL (Table 2) [22–24]. JPAC-QOL has been shown to have acceptable reliability and validity to be used for psychometric evaluation in patients complaining of functional constipation [25].

**Table 1:** Study endpoints

**Primary endpoint**	
• Change in JPAC-QOL from baseline at 2 weeks	
**Key secondary endpoints**	
• Change in SBMs from baseline at 2 and 12 weeks	
**Other secondary endpoints**	
• Change in JPAC-QOL from baseline at 12 weeks	
• Change in PAC-SYM from baseline at 2 and 12 weeks	
• Change in CSS from baseline at 2 and 12 weeks	
• Change in Rome IV from baseline at 2 and 12 weeks	
• Change in BSFS from baseline at 2 and 12 weeks	
• Change in SF-36 from baseline at 2 and 12 weeks	
**Safety endpoint**	
• Assessment of adverse events that appeared from days 1 to 28 after treatment	

*Abbreviations: BSFS* Bristol Stool Form Scale, *CSS* Constipation Scoring System, *JPAC-QOL* Japanese version of Patient Assessment of Constipation Quality of Life questionnaire, *PAC-SYM* Patient Assessment of Constipation Symptoms, *SBMs* Spontaneous bowel movements, *SF-36* 36-item Short Form Health Survey

**Table 2:** Japanese version of Patient Assessment of Constipation Quality of Life

The following questions are designed to measure the impact constipation has had on your daily life over the past 2 weeks. For each question, please check one box.					
**The following questions ask about your symptoms related to constipation. During the past 2 weeks, to what extent or intensity have you…**	**Not at all = 1**	**A little bit = 2**	**Moderately = 3**	**Quite a bit = 4**	**Extremely = 5**
1. Felt bloated to the point of bursting?	□	□	□	□	□
2. Felt heavy because of your constipation?	□	□	□	□	□
**The next few questions ask about how constipation affects your daily life. During the past 2 weeks, how much of the time have you…**	**None of the time = 1**	**A little of the time = 2**	**Some of the time = 3**	**Most of the time = 4**	**All of the time = 5**
3. Felt any physical discomfort?	□	□	□	□	□
4. Felt the need to have a bowel movement but not been able to?	□	□	□	□	□
5. Been embarrassed to be with other people?	□	□	□	□	□
6. Been eating less and less because of not being able to have bowel movements?	□	□	□	□	□
**The next few questions ask about how constipation affects your daily life. During the past 2 weeks, to what extent or intensity have you…**	**Not at all = 1**	**A little bit = 2**	**Moderately = 3**	**Quite a bit = 4**	**Extremely = 5**
7. Had to be careful about what you eat?	□	□	□	□	□
8. Had a decreased appetite?	□	□	□	□	□
9. Been worried about not being able to choose what you eat (for example, at a friend’s house)?	□	□	□	□	□
10. Been embarrassed about staying in the bathroom for so long when you were away from home?	□	□	□	□	□
11. Been embarrassed about having to go to the bathroom so often when you were away from home?	□	□	□	□	□
12. Been worried about having to change your daily routine (for example, traveling, being away from home)?	□	□	□	□	□
**The next few questions ask about your feelings related to constipation. During the past 2 weeks, how much of the time have you…**	**None of the time = 1**	**A little of the time = 2**	**Some of the time = 3**	**Most of the time = 4**	**All of the time = 5**
13. Felt irritable because of your condition?	□	□	□	□	□
14. Been upset by your condition?	□	□	□	□	□
15. Felt obsessed by your condition?	□	□	□	□	□
16. Felt stressed by your condition?	□	□	□	□	□
17. Felt less self-confident because of your condition?	□	□	□	□	□
18. Felt in control of your situation?	□	□	□	□	□
**The next questions ask about your feelings related to constipation. During the past 2 weeks, to what extent or intensity have you…**	**Not at all = 1**	**A little bit = 2**	**Moderately = 3**	**Quite a bit = 4**	**Extremely = 5**
19. Been worried about not knowing when you are going to be able to have a bowel movement?	□	□	□	□	□
20. Been worried about not being able to have a bowel movement?	□	□	□	□	□
21. Been increasingly bothered by not being able to have a bowel movement?	□	□	□	□	□
**The next questions ask about your life with constipation. During the past 2 weeks, how much of the time have you…**	**None of the time = 1**	**A little of the time = 2**	**Some of the time = 3**	**Most of the time = 4**	**All of the time = 5**
22. Been worried that your condition will get worse?	□	□	□	□	□
23. Felt that your body was not working properly?	□	□	□	□	□
24. Had fewer bowel movements than you would like?	□	□	□	□	□
**The next questions ask about your degree of satisfaction related to constipation. During the past 2 weeks, to what extent or intensity have you been…**	**Not at all = 1**	**A little bit = 2**	**Moderately = 3**	**Quite a bit = 4**	**Extremely = 5**
25. Satisfied with how often you have a bowel movement?	□	□	□	□	□
26. Satisfied with the regularity of your bowel movements?	□	□	□	□	□
27. Satisfied with the time it takes for food to pass through the intestines?	□	□	□	□	□
28. Satisfied with your treatment?	□	□	□	□	□

The secondary endpoints include the change of baseline JPAC-QOL scores at 12 weeks and change in Patient Assessment of Constipation-Symptoms, Constipation Scoring System, Rome IV criteria, Bristol Stool Form Scale, spontaneous bowel movements (SBMs), and 36-item Short Form Health Survey at 2 and 12 weeks after commencing the intervention.

### Dosing rationale

A Japanese multi-institutional retrospective study reported that prophylactic intake of 1000 to < 2000 mg/day magnesium oxide was significantly effective in preventing constipation during oral opioid therapy [26]; therefore, we chose a dose of 1500 mg (the median effective dose). Since the only permitted dose of naldemedine in Japan is 0.2 mg, we chose this dose for this trial.

### Drug supply

Both the doctor and patient will be aware of the treatment allocation. The doctor will prescribe magnesium oxide 1500 mg/day or naldemedine 0.2 mg/day according to the drug name provided by the patient enrollment center. To improve adherence to interventional protocols, patients will be required to return the unused tablets at the last visit, which will be counted and recorded in the medical records.

### Sample size estimation

Our retrospective analysis of magnesium oxide/naldemedine in 10 patients with OIC at Yokohama City University Hospital showed mean JPAC-QOL changes of − 1.19 and − 0.76 in the naldemedine and magnesium oxide groups, respectively. We decided to calculate the sample size required to conduct a proper analysis of variance F-test on the basis of these data. Assuming that mean changes in the JPAC-QOL score in the naldemedine and magnesium oxide groups would be − 1.19 and − 0.76, respectively, with a common standard deviation of 0.76, we determined that 51 patients would be needed in each group to reach 90% statistical power with a two-sided significance level of 5%. To compensate for any dropout, we proposed a sample size increase to 60 per group. To reach this sample size, a total of 120 patients will be needed in the study.

### Eligibility

The target study subjects are adult patients (20–85 years of age) with cancer who will commence opioid therapy for cancer pain. There is no distinction in the type and location of cancer. Type, dose, or frequency of opioid medication will not be restricted in this study. Eligible subjects will be required not to have used laxatives before the study intervention. If severe OIC that cannot be controlled by magnesium oxide or naldemedine occurs during the intervention, the use of senna will be permitted. The inclusion and exclusion criteria are presented in Table 3.

**Table 3:** Inclusion and exclusion criteria

**Inclusion criteria**	
Males and females 20–85 years of age	
Patients who have not started opioid therapy	
Patients who will commence opioid therapy for cancer pain	
Patients capable of oral intake	
Patients capable of reporting the patient-reported outcomes	
Patients who are expected to stay in stable pathological condition during the observation period	
Patients who are able to provide written consent to participate in this research, follow instructions during participation, undergo protocol-specified physical examinations and other examinations, and report their symptoms or events	
**Exclusion criteria**	
Patients with any contraindications listed on the package insert for magnesium oxide/naldemedine or with a history of hypersensitivity to any ingredients of these drugs	
Patients with a serious gastrointestinal structural anomaly (e.g., mechanical ileus), a disease that influences intestinal transit (e.g., paralytic ileus, peritoneal dissemination, peritoneal cancer, uncontrolled hyper-/hypothyroidism), irritable bowel syndrome, inflammatory bowel disease (e.g., ulcerative colitis, Crohn disease), active diverticular disease, pelvic disorders that cause constipation (e.g., uterine prolapse, rectal prolapse, myoma of the uterus that influences defecation), or patients whom the doctor decides have conditions with serious influence on gastrointestinal function (e.g., difficulty with oral intake), even if the aforementioned diseases are cured	
Breastfeeding women or women with possible pregnancy	
Patients who have undergone a surgery or a treatment that influences gastrointestinal function (e.g., nerve block) within 28 days before the enrollment day or patients planning to undergo go such surgery or treatment during the observation period	

### Randomization and masking

Eligible patients satisfying the screening inclusion and exclusion criteria will be invited to participate in the study by the investigators. Patients will be randomly assigned in a 1:1 ratio to receive 500 mg of magnesium oxide thrice daily or 0.2 mg of naldemedine once daily at the central registration center. Randomization will be performed after the patient has signed the informed consent form. The principal investigator or coinvestigator will be notified of the patient identification number and drug name by fax from the patient enrollment center. To avoid a large bias, we will stratify patients by age (< 65 or ≥ 65 years) and sex (male or female) using a computer-generated administered procedure with a permuted block method at an independent institution. Masking of patients and physicians is not applicable, because this is an open-label study, but the independent outcome evaluator will be masked to treatment assignments.

### Adverse event monitoring

The investigators will be required to record all adverse events (AEs) that occur during the study in the medical records, including information about onset and end date (if applicable), AE severity and seriousness, the investigator’s opinion of the association with magnesium oxide or naldemedine treatment, action taken regarding magnesium oxide or naldemedine use and AE treatment, cause of event (if known), and information regarding the resolution or outcome. AEs classified as serious will be recorded using a serious AE reporting tool. The intensity of an AE will be graded according to the National Cancer Institute Common Terminology Criteria for Adverse Events (NCI-CTCAE) version 4.0, which includes the classifications of AE intensity shown in Table 4. Any abnormal results related to study drug treatment will be reported weekly until the abnormality is resolved or otherwise explained.

**Table 4:** Adverse events

Grade	Description
Grade 1 (mild)	Asymptomatic or mild symptoms, clinical or diagnostic observations only, intervention not indicated
Grade 2 (moderate)	Minimal, local, or noninvasive intervention indicated, limiting age-appropriate instrumental ADL
Grade 3 (severe)	Medically significant but not immediately life-threatening, hospitalization or prolongation of hospitalization indicated, disabling, limiting self-care ADL
Grade 4 (life-threatening)	Life-threatening consequences, urgent intervention indicated
Grade 5 (death)	Death related to AE

*ADL* Activities of daily living, *AE* Adverse events

### Criteria for discontinuation

Study treatment will be discontinued when a grade 3 or higher severe AE according to the NCI-CTCAE version 4.0 occurs, when oral compliance is < 80%, or when a patient is found to be ineligible for the trial. Treatment will also discontinue if requested by a patient or if continuous medical examination becomes challenging because of patient relocation, change in hospital or business, or discontinuation of the study.

### Definition of protocol deviations

Protocol deviations are defined as follows:

1. Dropout before randomization: patients who were not randomized after informed consent
2. Screen failure: patients who do not meet the inclusion criteria or who do meet the exclusion criteria
3. Patients who were not treated: patients who did not receive the study drugs
4. Fulfillment of criteria for discontinuation: patients who met the criteria described in “criteria for discontinuation” but did not discontinue the study treatment or who did not meet the criteria but discontinued the study treatment during the observation period
5. Nonadherence to dosage regimen: patients with any deviation from the protocol relating to the dosage regimen
6. Violation of concomitant medications/therapy requirement: patients who had concomitant medications (therapy) that were prohibited in the protocol
7. Violation of the methods or timing of observations, tests, or assessments requirement: patients with any deviation from the protocol relating to the methods or timing of observation, test, or assessment

### Efficacy evaluation

JPAC-QOL score (the primary endpoint) will be calculated as the mean of the difference from baseline at 2 weeks. The secondary efficacy endpoints will be calculated as the mean of the difference from baseline at 2 or 12 weeks.

### Safety evaluation

AEs, dropout ratios, and physical examinations are the chosen safety evaluations of this trial. Physical assessments will be performed and analyzed using standard procedures in Yokohama City University. Dropout will be defined as oral compliance < 80%.

### Statistical hypothesis

The full analysis set is defined as all patients who receive any amount of the study medication with initial information on the primary endpoint. The full analysis set will be the primary analysis set for efficacy to use as an intention-to-treat patient population. For the primary endpoint, one-way analysis of variance will be performed between the two groups to calculate the *p* value using Student’s *t* test. The *p* value will be significant at a two-sided significance level of 5%, and both the *p* value and confidence intervals will be used to determine the statistical significance of our results. The paired *t* test or Wilcoxon signed-rank test will be performed for within-group comparisons before and after the intervention. The chi-square test will be used to assess the frequency of AEs, and the treatment compliance rate will be calculated and compared using Fisher’s exact test. JMP version 11.2.0 software (SAS Institute, Cary, NC, USA) will be used for all statistical analyses. Complete case analysis based on likelihood will be used for the primary analysis, or a multiple imputation method will also be used to handle missing data as a sensitivity analysis.

### Trial steering and data monitoring committees

The trial steering and independent data monitoring committees will be located at the Department of Biostatistics, Yokohama City University School of Medicine and Yokohama City University Center for Novel and Exploratory Clinical Trials. The management team will conduct on-site monitoring and meet with the facility person in charge when necessary. Any visit to the facility will be reported in the monitoring report.

In principle, the first patient will be monitored continuously throughout the trial, and, if there is no problem, every 10th patient will be monitored. To confirm that necessary documents are stored properly, on-site monitoring will be performed appropriately, and, if there are any problems, corrective action will be taken. The result will be recorded in the monitoring report. The data monitoring committee will have access to the final trial dataset, and there is no contractual agreement regarding investigators’ access restrictions to the dataset.

## Discussion

Patients with OIC report a significantly worse QOL than those who are unaffected by OIC [9, 21], owing to associated symptoms such as abdominal pain, sensation of fullness, and loss of appetite, but their QOL improves after symptom resolution [23]. JPAC-QOL is a reliable method for measuring the QOL of patients with constipation. A decrease in constipation can also be determined by the number of times a patient defecates using the SBM score, but evaluating patient comfort solely using this objective index is challenging because of the high interindividual differences in defecation times. QOL improvement is particularly important in patients with cancer, and thus we chose change in JPAC-QOL score as the primary endpoint of this study.

In this study, we chose magnesium oxide as the control because in Japan, its preventive intake is reported to dampen OIC when patients eventually commence opioid therapy [16], and osmotic laxatives, including magnesium oxide, are conventionally used to treat OIC. Other laxatives, such as senna, lactulose, and sodium picosulfate hydrate, are also used, and all are effective. However, a systematic review by Miles et al. [27] indicated no evidence of superiority of one laxative or specific combination of laxatives for the management of constipation in palliative care patients. Similarly, Agra et al. [28] reported no difference in the effects of senna and lactulose after observing the subjective index for over 72 h and the number of days with defecation throughout the study.

Magnesium oxide is conventionally used for OIC prevention in Japan; therefore, its long-term safety is empirically established. In addition, magnesium oxide has advantages in terms of medical cost at 33.6 yen/day (1500 mg/day) over naldemedine, which costs 272.1 yen/day. Naldemedine may have the advantage of adherence with a once-daily required intake.

A good number of OIC treatment studies exist, with only a few on the use of preventive laxatives against OIC. Additionally, some limitations of our study are that it is conducted at a single center, its open-label design, and a potentially short treatment period (12 weeks). The rationale for conducting this trial as an open-label study is as follows:

1. This trial compares two drugs, which are already on the market and used in clinical practice.
2. Both agents compared in this trial are active drugs.
3. The double-dummy method is required for blinding, and the logistics of using that method in a study, such as placebo manufacturing costs, drug management, and drug dispensing, are challenging.
4. This is an exploratory study.

We have considered the need to blind the next phase using the double-dummy method. Further research is encouraged.

## Dissemination

The results of this study will be submitted for publication in international peer-reviewed journals, and the key findings will be presented at conferences. Authorship will be ascribed in accordance with the International Committee of Medical Journal Editors guidelines.

## Trial status

Protocol version: 1.0, November 26, 2017. Recruitment began on March 22, 2018, and was ongoing as of May 26, 2020.

## Data Availability

Not applicable**.**

## Abbreviations

ADL: Activities of daily living
AE: Adverse events
BSFS: Bristol Stool Form Scale
CSS: Constipation Scoring System
JPAC-QOL: Japanese version of Patient Assessment of Constipation Quality of Life questionnaire
NCI-CTCAE: National Cancer Institute Common Terminology Criteria for Adverse Events
OIC: Opioid-induced constipation
PAC-SYM: Patient Assessment of Constipation Symptoms
PAMORA: Peripherally acting μ-opioid receptor antagonist
QOL: Quality of life
SBM: Spontaneous bowel movement
SF-36: 36-Item Short Form Health Survey
